# Exploration of the Immuno-Inflammatory Potential Targets of Xinfeng Capsule in Patients with Ankylosing Spondylitis Based on Data Mining, Network Pharmacology, and Molecular Docking

**DOI:** 10.1155/2022/5382607

**Published:** 2022-03-23

**Authors:** Yanyan Fang, Jian Liu, Ling Xin, Jianting Wen, Jinchen Guo, Dan Huang, Xu Li

**Affiliations:** ^1^The First Affiliated Hospital of Anhui University of Chinese Medicine, Hefei, Anhui 230038, China; ^2^Key Laboratory of Xin'an Medicine of the Ministry of Education, Anhui University of Chinese Medicine, Hefei, Anhui 230038, China; ^3^Anhui University of Chinese Medicine, Hefei, Anhui 230031, China

## Abstract

**Objective:**

This study aimed to ascertain the immuno-inflammatory molecular targets of Xinfeng capsules (XFC) in the treatment of ankylosing spondylitis (AS) based on data mining, network pharmacology, and molecular docking.

**Methods:**

The efficacy of XFC in the treatment of AS was assessed by clinical data mining. Network pharmacology was utilized to establish a network of the targets for XFC active ingredients in the treatment of AS. The binding mode and affinity of XFC active ingredients to the key targets for AS were predicted using molecular docking.

**Results:**

XFC significantly diminished immuno-inflammatory indicators of AS. In total, 208 targets of XFC were obtained from the TCMSP database and 629 disease targets of AS were screened from the GeneCards database, which were intersected to yield 57 targets of XFC in the treatment of AS. Protein-protein interaction, gene ontology, and Kyoto genome encyclopedia analyses showed that XFC might activate TNF and NF-*κ*B signaling pathways. Quercetin, kaempferol, triptolide, and formononetin had free binding energies < -9 kcal/mol to inflammatory targets (TNF and PTGS2) in the molecular docking analysis of XFC-active ingredients, indicating that TNF and PTGS2 might be the targets of the action of XFC.

**Conclusions:**

Collectively, XFC had a significant therapeutic effect on AS. Specifically, the active ingredients of XFC, including quercetin, kaempferol, triptolide, and formononetin, inhibited the inflammatory response in AS by downregulating TNF and PTGS2 in the TNF and NF-*κ*B signaling pathways.

## 1. Introduction

Ankylosing spondylitis (AS) is a kind of chronic inflammatory arthritis that mainly afflicts the axial and sacroiliac joints [1]. It occurs more frequently in men with a high incidence and disability rate [2, 3]. If AS is not treated appropriately, this inflammatory damage normally causes chronic back pain and morning stiffness in patients, thereby resulting in the restriction of spinal mobility and functional impairment status of patients [4–6], which seriously affect the life quality of patients. According to existing research, the pathogenesis of AS is related to many factors. However, the specific etiology is still unclear [7]. At present, the most common clinical treatments for AS mainly include symptomatic treatments to relieve the pain of patients. However, the effects of these treatments are not ideal and often lead to certain side effects [8]. Therefore, the development of new AS treatment strategies is essential.

Xinfeng capsules (XFC), as an in-hospital preparation of the First Affiliated Hospital of Anhui University of Traditional Chinese Medicine (TCM), has the effects of invigorating spleens, replenishing Qi, removing dampness and numbness, and activating blood and collaterals [9]. The previous study has proved the safe and controllable curative effects of XFC in the clinical application of AS [10].

Data mining technology is a technology that explores potentially valuable knowledge from a large amount of data, which has been widely used in the medical domain [11]. Retrospective data mining research can find meaningful results from clinical data. Network pharmacology technology has been utilized to predict the mechanism behind the action of drugs by constructing a complex network among drugs, ingredients, targets, diseases, and pathways, which is suitable for the studies of complex system mechanisms of TCM [12].

This piece of research sets out to screen the chemical active ingredients and disease-related targets of XFC in AS treatment on the basis of the methods of clinical data mining, network pharmacology, and molecular docking through data analysis under the guidance of bioinformatics theory to assess the impacts of the treatment process. The enrichment analysis of the highly correlated targets was conducted using molecular docking technology to fit the binding activity between the drug-active ingredients and target protein molecules to explore the potential effective ingredients and molecular mechanism of XFC in the treatment of AS and to further clinically verify the mechanism underlying the action of XFC.

## 2. Materials and Methods

### 2.1. Acquisition of Clinical Data and Analysis of Association Rules

The inpatient data were collected for 908 patients who were AS inpatients between July 2009 and December 2020 in the Department of Rheumatology and Immunology of the First Affiliated Hospital of Anhui University of Chinese Medicine. The data included the usage of XFC and the immuno-inflammatory indexes, such as the erythrocyte sedimentation rate (ESR), C-reactive protein (CRP), immunoglobulin A (IgA), immunoglobulin *M* (IgM), immunoglobulin *G* (IgG), complement component 3 (C3), and C4. This study was approved by the Medical Ethics Committee of Anhui Provincial Hospital of Traditional Chinese Medicine (2020AH-08). All study participants provided the written informed consent. The Apriori module in the IBM SPSS Modeler 18.0 software was adopted to analyze the correlation between XFC and immune-inflammatory indicators. The formula is as follows [13]:

(1) $$\begin{array}{c} Support\left(X⟶Y\right)=\frac{\left(X\cup Y\right)}{N},\\ Confidence\left(X⟶Y\right)=\frac{\left(X\cup Y\right)}{\sigma \left(X\right)},\\ Lift\left(X⟶Y\right)=Confidence\frac{\left(X⟶Y\right)}{\sigma \left(Y\right)}. \end{array}$$

### 2.2. Acquisition and Screening of Pharmaceutical Ingredients

The chemical active ingredients of *Astragalus*, coix seed, *Tripterygium wilfordii*, and centipede in the XFC prescription were acquired from the traditional Chinese medicine system pharmacological analysis platform (TCMSP, http://tcmspw.com/) [14]. The above ingredients were retrieved as per the toxic pharmacokinetic parameters (absorption, distribution, metabolism, and excretion) [15] with the screening criteria of the oral availability of the drug ≥30% and the drug similarity ≥0.18 [16, 17]. The relevant literature was employed to improve the data.

### 2.3. Prediction of Drug Component Targets and Acquisition of Disease-Related Targets

The targets of the active ingredients of XFC were obtained using the predicted targets of the active ingredients in drugs from the TCMSP database. The predicted targets of the chemical active ingredients were harvested after the completion of the standardization conversion in the Universal Protein Resource (UniProt). Then, the network visualization software, Cytoscape [18], was applied to construct and analyze the network characteristics and the targets of the active ingredients to clarify the relationship between the active ingredients and the targets of XFC.

With “AS” utilized as a search term, the disease-related targets were screened in the databases of GeneCards (https://www.genecards.org/) and OMIM (http://www.omim.org/). The data of these two databases were intersected, and the unique values were retained to obtain known disease-related targets of AS.

### 2.4. Protein-Protein Interaction (PPI) Network Construction and Analysis

The overlapping targets were imported into the STRING [19] protein interaction database (http:/string-db.org/) with the species defined as “*Homo sapiens*” and the confidence value set as ≥ 0.4, followed by the PPI network analysis of the overlapping targets. The acquired data were imported into the Cytoscape software to analyze the network topology parameter value, thus screening the top ten targets of the degree value (DC value) as the key targets in the network.

### 2.5. Pathway Enrichment Analysis

The overlapping targets obtained by the PPI network analysis were uploaded to the database for annotation, visualization, and integrated discovery (David) [20], followed by gene ontology (GO) function and Kyoto Encyclopedia of Genes and Genomes (KEGG) pathway enrichment analyses with the condition value as *p* < 0.05. The top 10 pathways in the enrichment analysis were selected to draw a bubble chart.

### 2.6. Verification of Molecular Docking between Key Targets and Pharmaceutical Ingredients

The key targets (top 10 target proteins) were selected in accordance with the median value of the PPI network. Thereafter, the corresponding chemical active ingredients related to these target proteins were identified in the protein interaction network to confirm the binding activity of these key target proteins to the chemical active ingredients.

The TCMSP database was applied to acquire the chemical structure of the chemical active ingredients. The structure files of the target protein were identified in the RCSB PDB database (http://www.rcsb.org/) with the species restricted to “*Homo sapiens.*” We preferred the proteins with original ligand small molecules and high similarity between small molecular structure and the active ingredients of the docking chemical. The Autodock Vina was employed to perform modification operations, such as dehydration and hydrogenation, on the retrieved target protein. The protein was output in PDBQT format, and molecular docking was implemented to determine the global optimal binding conformation according to the value of docking binding energy. PyMOL was utilized to visualize the docking result.

### 2.7. Statistical Analysis

The data were analyzed using the GraphPad Prism 8 software. Continuous variables were analyzed by *t*-test. *p* < 0.05 was statistically different.

## 3. Results

### 3.1. Changes in Immune-Inflammatory Indexes after XFC Treatment

To observe the situation of the patients with AS after XFC treatment, we selected clinical immune-inflammatory indexes for validation, including ESR, CRP, IgA, IgM, IgG, C3, and C4. Compared with the indexes before treatment, the levels of ESR, CRP, IgA, IgG, C3, and C4 were significantly decreased (*p* < 0.01; Table 1), while there was no significant change in IgM.

### 3.2. Analysis of Association Rules between XFC and Immune-Inflammatory Indexes

The minimum confidence degree was set to 50%, and the minimum support degree was set to 10%. The correlation between XFC and immune-inflammatory indexes was acquired using the Apriori module. Association rule analysis showed that the confidence degree of XFC with improvement on CRP was 78.23%. The confidence degree with improvement on ESR was 63.79%, and the confidence degree with improvement on IgA was 66.67%. Meanwhile, all the lifting degrees are greater than 1. They indicated that XFC was strongly associated with the improvement of AS immune-inflammatory indexes. The results were shown in Table 2.

### 3.3. Potential Active Ingredients of XFC Drugs

A total of 92 chemical active ingredients in the XFC formula were harvested from the TCMSP database, among which 20 were *Astragalus*, 9 were coix seed, 51 were *Tripterygium wilfordii*, and 12 were centipede (Supplementary Table S1).

### 3.4. The Prediction of the Targets of XFC-Active Ingredients

**Figure 1:**
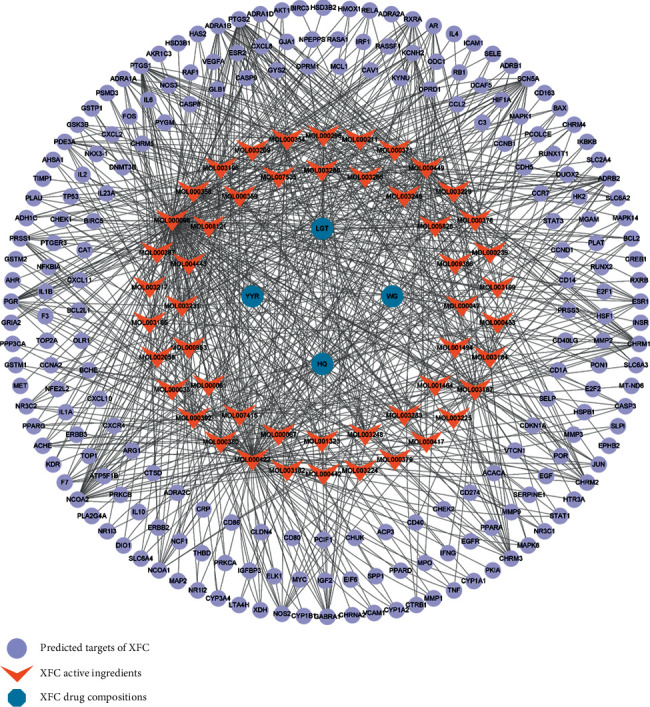
The active ingredient-target network diagram of XFC.

The target prediction function of the TCMSP database was utilized. Subsequent to data screening and duplicate value removal, 208 predicted targets of chemical active ingredients in the XFC formula were yielded. The Cytoscape software was employed to complete the relationship network diagram of the effective chemical ingredients and predict the targets of the XFC formula (Figure 1). Among them, there were 818 interaction relationships and 263 targets for active ingredients and targets.

### 3.5. The Disease-Related Targets of AS

**Figure 2:**
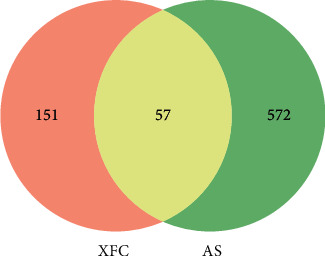
The Venn diagram of XFC targets and disease-related targets. *Note.* XFC stands for XFC targets, and AS stands for disease-related targets.

The targets obtained in each database were intersected, screened, and processed through the GeneCards database, which yielded 629 disease-related targets of AS. The Venn diagram of “drug-disease-related target” (Figure 2) was plotted, which acquired 57 targets of XFC for AS treatment (Supplementary Table S2).

### 3.6. PPI Network Analysis Results

**Figure 3:**
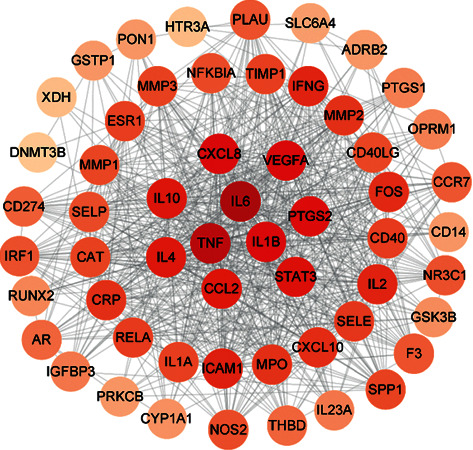
The PPI network of XFC in the treatment of AS. *Note:* the darker the color, the higher the degree value.

The PPI network diagram of XFC in AS treatment (Figure 3) had 56 nodes and 704 interaction relationships. The target proteins were ranked based on the degree value (DC value), and the top 10 target proteins were selected, including IL-6, TNF, VEGFA, CXCL8, IL-1B, PTGS2, STAT3, CCL2, IL-10, and IL-4. These 10 proteins were defined as the key targets in the PPI network diagram (Table 3).

### 3.7. Pathway Enrichment Analysis Results

**Figure 4:**
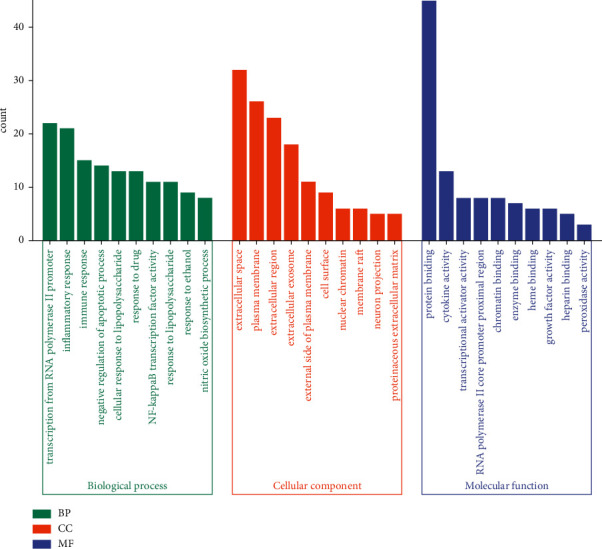
The results of GO function enrichment analysis.

The enrichment analysis of overlapping targets (57 targets of XFC for AS treatment) was implemented using the David database, demonstrating that the overall treatment process had multiple signaling pathways that participated in the orchestration of various biological processes. The results of the GO function enrichment analysis displayed that the overlapping targets of XFC in AS treatment were mainly enriched in biological processes, such as inflammatory response, cell response to lipopolysaccharide, positive regulation of NF-*κ*B transcription factor activity, and immune response. These overlapping targets were majorly enriched in cell components, including extracellular space, extracellular zone, outer plasma membrane, plasma membrane, cell surface, nuclear chromatin, membrane raft, extracellular exosomes, neuron projection, and protein extracellular matrix. Additionally, these overlapping targets were mostly enriched in molecular functions, such as cytokine activity, transcription activator activity, RNA polymerase II core promoter proximal region sequence-specific binding, and protein binding (Figure 4).

**Figure 5:**
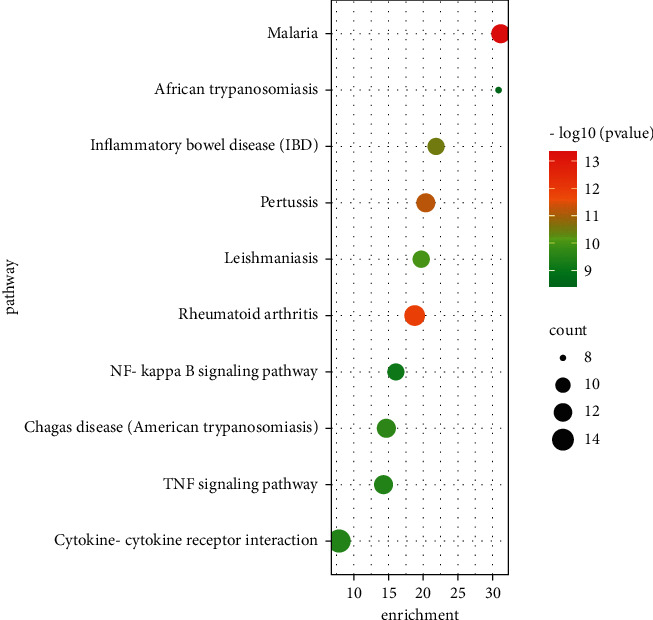
The results of KEGG pathway enrichment analysis.

KEGG enrichment analysis manifested that the related targets were significantly enriched in signaling pathways, such as TNF signaling pathway, NF-*κ*B signaling pathway, and cytokine-cytokine receptor interaction (Figure 5).

### 3.8. The Results of Molecular Docking

**Figure 6:**
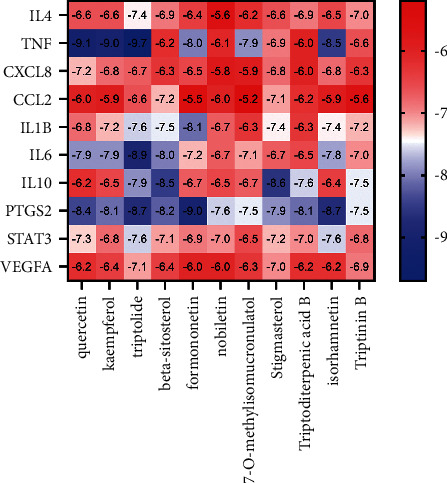
The binding energy of chemical active ingredients to the key target proteins.

**Figure 7:**
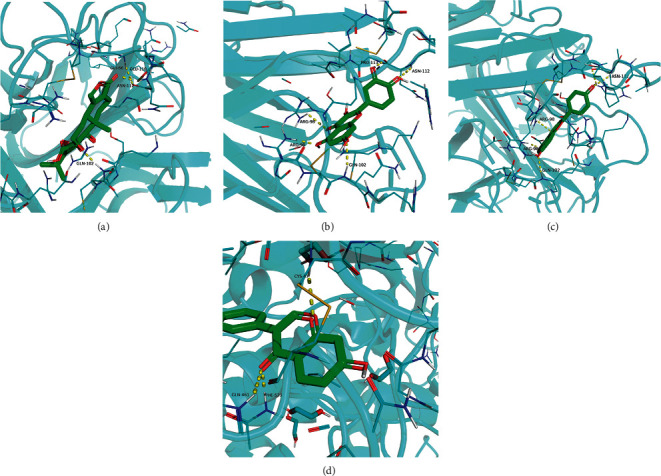
The docking pattern diagram of the inflammatory targets in the network and the lowest component of their binding energy. *Note.* (a) The docking diagram of triptolide (MOL003187) and TNF (2e7a). (b) The docking diagram of quercetin (MOL000098) and TNF (2e7a). (c) The docking diagram of kaempferol (MOL000422) and TNF (2e7a). (d) The docking diagram of formononetin (MOL000392) and PTGS2 (5f19).

The key targets in the PPI network were selected, and the relevant components in reverse were identified in the light of the protein interaction network for molecular docking. When the binding energy between the ligand and the receptor was lower, the binding conformation was more stable and the possibility of interaction was greater. The validation of the molecular docking results documented that the binding energy of the effective chemical active ingredients to the key target proteins was less than −5 kcal/mol (Figure 6), suggesting that the binding activity of the compound to the receptor was stable. The PyMOL software was applied to visualize the protein docking pattern of the top four target proteins with binding energy (<-9 kcal/mol) and the anti-inflammatory active ingredients (Figure 7).

## 4. Discussion

There was no record of the disease name in ancient medical records about AS, and AS belongs to the categories of “*Bi syndrome*”, “*Gubi*”, “*Shenbi*”, and “*Dalv*” according to the clinical manifestations [21]. AS mainly results from the invasion of external pathogens, such as wind, cold, and dampness. At the same time, the insufficiency in the congenital endowment or the acquired loss of nutrition results in the deficiency of spleen Qi in patients, and thus the body system is imbalanced because of insufficient righteousness to resist external pathogens to weaken the function of spleen deficiency and dampness. AS contributes to the mutual accumulation of phlegm and blood stasis, unclear blood stasis, back pain, and spinal rigidity. Hence, TCM believes that the pathogenesis of AS is attributable to the deficiency of spleen Qi and blood stasis [22]. XFC has been documented to assume a good therapeutic role in the treatment of AS. However, because of the numerous chemical active ingredients in TCM and the lack of molecular research, the action mechanism of TCM in the treatment process remains unclear. Based on network pharmacology, bioinformatics analysis, and molecular docking technology, this study analyzed the active ingredients, the potential targets, and the pathways of XFC in the treatment of AS to study its molecular mechanism.

Our results elucidated that XFC could remarkably improve the immune-inflammatory indicators of AS and that the treatment of AS by XFC might act on IL-6, TNF, VEGFA, CXCL8, IL-1B, PTGS2, STAT3, CCL2, IL-10, and IL-4 through chemical active ingredients, such as quercetin, kaempferol, triptolide, *β*-sitosterol, formononetin, cerebellin, 7-O-Methyl isorhamnitol, phytosterol, triterpene acid B, and isorhamnetin. Thus, XFC orchestrated the biological processes involved in inflammation (cell response to lipopolysaccharide, positive regulation of NF-*κ*B transcription factor activity, and immune response) and signaling pathways (TNF signaling pathway, NF-*κ*B signaling pathway, and cytokine-cytokine receptor interaction), thus exerting a multicomponent, multitarget, and multinetwork anti-inflammatory pharmacological effect.

The active ingredients with high values in the “drug-component-target” network included quercetin, kaempferol, triptolide, and formononetin. Quercetin, belonging to natural flavonols, has various effects, such as anti-inflammation, anti-platelet aggregation, and anti-oxidation. Quercetin inhibits inflammation by downregulating proinflammatory factors and upregulating anti-inflammatory factors [23–25]. Triptolide can obviously repress the expression of proinflammatory factors (TNF-*α* and IL-6) stimulated by lipopolysaccharide to exert an anti-inflammatory effect. The mechanism underlying the action of triptolide is to promote I*κ*B upregulation and suppress I*κ*B*α* phosphorylation, thereby blocking nuclear NF-*κ*B p65 activation [26]. Kaempferol is a flavonoid compound that has a variety of biological functions, such as anti-inflammatory, antitumor, antioxidant, and immunosuppressive activities, as well as the inhibition of IL-1*β*, IL-6, IL-8, and TNF-*α* levels, thus playing an anti-inflammatory role [27, 28]. Formononetin is an effective ingredient in the legume red clover, which can effectively restrain the inflammatory response in the pathological process of numerous diseases [29]. Inflammation is the key factor leading to the clinical symptoms of AS, such as joint damage, disability, and comorbidities. Hence, anti-inflammation is the primary treatment strategy [30]. The major pharmacological effects of the active ingredients are relatively concordant with the anti-inflammatory mechanism of AS.

The results of the GO function enrichment analysis reflected that the treatment process was highly correlated with inflammation, cell response to lipopolysaccharide, positive regulation of NF-*κ*B transcription factor activity, and immune response. Among them, the inflammatory response is strongly associated with the pathogenesis of AS.

In the results of the KEGG pathway enrichment analysis, the XFC formula exerted anti-inflammatory effects in AS by manipulating the TNF signaling pathway, NF-*κ*B signaling pathway, and cytokine-cytokine receptor interaction. TNF in the TNF signaling pathway and NF-*κ*B signaling pathway is one of the cytokines in the acute phase of inflammation, which is implicated in the systemic inflammatory response, and it accelerates the production of interleukins. TNF can repress chondrocyte apoptosis and cartilage bone destruction by inactivating TNF-binding receptors to reduce matrix metalloproteinase [31, 32]. PTGS2 is rich in the TNF and NF-*κ*B signaling pathways. The official website of KEGG unraveled that PTGS2 in these two signaling pathways modulated inflammation. PTGS2 upregulation can lead to the proliferation of fibroblast-like synovial cells, which influences the levels of TNF-*α*, IL-1*β*, and IL-6 in the serum and aggravates the inflammatory response [33]. Moreover, triptolide selectively suppresses COX-2 expression in human synovial fibroblasts, and the production of PGE2 inhibits IL-1*α* and diminishes TNF-*α* expression in the synovium of rats with adjuvant arthritis induced by collagen [34, 35].

The results of molecular docking unveiled that the docking energy of the key targets in the PPI network and their corresponding chemical active ingredients were all less than −5 kcal/mol, indicating that the chemical active ingredients in the XFC formula and the disease-related targets of AS produced better protein-binding activity. In addition, the binding energies of quercetin, kaempferol, and triptolide to TNF-*α* and of formononetin to PTGS2 molecule docking did not exceed -9 kcal/mol. This result illustrated that the chemical active ingredients of the XFC formulas had excellent binding activity to inflammatory targets.

This article still has certain limitations. This article still lacks experimental verification although the mechanism behind the action of Chinese herbal compounds in the treatment of AS was preliminarily predicted and molecular docking verification was carried out.

## 5. Conclusions

This research was conducted based on the methodologies of network pharmacology and molecular docking. Through the evaluation of the pharmacokinetic parameters of the XFC formula components and the enrichment analyses, it was noted that XFC could orchestrate cytokine activity, transcription activator activity, RNA polymerase II core promoter proximal region sequence-specific binding, and protein binding. The molecular mechanism of XFC in the treatment of AS was mainly achieved by the active ingredients, including quercetin, kaempferol, and triptolide. Formononetin reduced the expression of inflammatory factors (TNF and PTGS2) via TNF and NF-*κ*B signaling pathways, thereby restricting the inflammatory response in AS.

## Figures and Tables

**Table 1:** Changes of inflammatory and immune indexes after XFC treatment.

	Before treatment	After treatment	*p* value
ESR (mm/h)	30.00 (15.00, 51.00)	21.00 (11.00, 39.00)	≤0.001
CRP (mg/L)	21.03 (6.43, 43.91)	8.24 (1.91, 22.98)	≤0.001
IgA (g/L)	2.50 (1.84, 3.37)	2.42 (1.75, 3.23)	≤0.001
IgM (g/L)	1.13 (0.86, 1.46)	1.15 (0.86, 1.48)	0.135
IgG (g/L)	12.78 (10.55, 15.52)	12.36 (10.20, 14.86)	≤0.001
C3 (g/L)	123.40 (87.40, 141.88)	114.10 (74.60, 132.55)	≤0.001
C4 (g/L)	28.40 (16.68, 35.80)	24.35 (13.45, 31.20)	≤0.001

*Note.* ESR, erythrocyte sedimentation rate; CRP, C-reactive protein; IgA, immunoglobulin A; IgM, immunoglobulin *M*; IgG, immunoglobulin *G*; C3, complement component 3; C4, complement component 4. *P* is the comparison between before and after treatment.

**Table 2:** Association rules of XFC with immune-inflammatory indexes.

Items (LHS ⇒ RHS)	Support	Confidence	Lift
{XFC} ⇒ {CPR}	17.32%	78.23%	1.05
{XFC} ⇒ {ESR}	16.20%	63.79%	1.06
{XFC} ⇒ {IgA}	13.79%	66.67%	1.02

**Table 3:** The key targets of XFC in the treatment of AS (the top 10 nodes ranked by value).

Number	Target protein name	Degree
1	IL-6	51
2	TNF	49
3	VEGFA	45
4	CXCL8	45
5	IL-1B	45
6	PTGS2	44
7	STAT3	42
8	CCL2	41
9	IL-10	40
10	IL-4	40

## Data Availability

All relevant data are included in the manuscript and supplementary material.

## Supplementary Materials

Supplementary Materials Active ingredients of XFC (Supplementary Table S1) and 57 targets of XFC for AS treatment (Supplementary Table S2) are in Supplemental Files.
