# Supplementary material for: Exploration of the Immuno-Inflammatory Potential Targets of Xinfeng Capsule in Patients with Ankylosing Spondylitis Based on Data Mining, Network Pharmacology, and Molecular Docking
Source: Evid Based Complement Alternat Med. 2022 Mar 23;2022:5382607. doi: 10.1155/2022/5382607 (PMC8967514; doi:10.1155/2022/5382607)
Supplement: Supplementary Materials — Active ingredients of XFC (Supplementary Table S1) and 57 targets of XFC for AS treatment (Supplementary Table S2) are in Supplemental Files. [file 5382607.f1.zip › 5382607.f1/Supplementary Table S1.docx]

Supplementary Table S1 Active ingredients of XFC

| **MOL ID** | **Active ingredient Name** | **Drug** |
| --- | --- | --- |
| MOL000211 | Mairin | Astragalus |
| MOL000239 | Jaranol | Astragalus |
| MOL000296 | hederagenin | Astragalus |
| MOL000033 | (3S,8S,9S,10R,13R,14S,17R)-10,13-dimethyl-17-[(2R,5S)-5-propan-2-yloctan-2-yl]-2,3,4,7,8,9,11,12,14,15,16,17-dodecahydro-1H-cyclopenta[a]phenanthren-3-ol | Astragalus |
| MOL000354 | isorhamnetin | Astragalus |
| MOL000371 | 3,9-di-O-methylnissolin | Astragalus |
| MOL000374 | 5'-hydroxyiso-muronulatol-2',5'-di-O-glucoside | Astragalus |
| MOL000378 | 7-O-methylisomucronulatol | Astragalus |
| MOL000379 | 9,10-dimethoxypterocarpan-3-O-β-D-glucoside | Astragalus |
| MOL000380 | (6aR,11aR)-9,10-dimethoxy-6a,11a-dihydro-6H-benzofurano[3,2-c]chromen-3-ol | Astragalus |
| MOL000387 | Bifendate | Astragalus |
| MOL000392 | formononetin | Astragalus |
| MOL000398 | isoflavanone | Astragalus |
| MOL000417 | Calycosin | Astragalus |
| MOL000422 | kaempferol | Astragalus |
| MOL000433 | FA | Astragalus |
| MOL000438 | (3R)-3-(2-hydroxy-3,4-dimethoxyphenyl)chroman-7-ol | Astragalus |
| MOL000439 | isomucronulatol-7,2'-di-O-glucosiole | Astragalus |
| MOL000442 | 1,7-Dihydroxy-3,9-dimethoxy pterocarpene | Astragalus |
| MOL000098 | quercetin | Astragalus |
| MOL000296 | hederagenin | Tripterygium wilfordii |
| MOL003182 | (+)-Medioresinol di-O-beta-D-glucopyranoside_qt | Tripterygium wilfordii |
| MOL003184 | 81827-74-9 | Tripterygium wilfordii |
| MOL003185 | (1R,4aR,10aS)-5-hydroxy-1-(hydroxymethyl)-7-isopropyl-8-methoxy-1,4a-dimethyl-4,9,10,10a-tetrahydro-3H-phenanthren-2-one | Tripterygium wilfordii |
| MOL003187 | triptolide | Tripterygium wilfordii |
| MOL003188 | Tripchlorolide | Tripterygium wilfordii |
| MOL003189 | WILFORLIDE A | Tripterygium wilfordii |
| MOL003192 | Triptonide | Tripterygium wilfordii |
| MOL003196 | Tryptophenolide | Tripterygium wilfordii |
| MOL003198 | 5 alpha-Benzoyl-4 alpha-hydroxy-1 beta,8 alpha-dinicotinoyl-dihydro-agarofuran | Tripterygium wilfordii |
| MOL003199 | 5,8-Dihydroxy-7-(4-hydroxy-5-methyl-coumarin-3)-coumarin | Tripterygium wilfordii |
| MOL003206 | Canin | Tripterygium wilfordii |
| MOL003208 | Celafurine | Tripterygium wilfordii |
| MOL003209 | Celallocinnine | Tripterygium wilfordii |
| MOL003210 | Celapanine | Tripterygium wilfordii |
| MOL003211 | Celaxanthin | Tripterygium wilfordii |
| MOL003217 | Isoxanthohumol | Tripterygium wilfordii |
| MOL003222 | Salazinic acid | Tripterygium wilfordii |
| MOL003224 | Tripdiotolnide | Tripterygium wilfordii |
| MOL003225 | Hypodiolide A | Tripterygium wilfordii |
| MOL003229 | Triptinin B | Tripterygium wilfordii |
| MOL003231 | Triptoditerpenic acid B | Tripterygium wilfordii |
| MOL003232 | Triptofordin B1 | Tripterygium wilfordii |
| MOL003233 | Triptofordin B2 | Tripterygium wilfordii |
| MOL003234 | Triptofordin C2 | Tripterygium wilfordii |
| MOL003235 | Triptofordin D1 | Tripterygium wilfordii |
| MOL003236 | Triptofordin D2 | Tripterygium wilfordii |
| MOL003238 | Triptofordin F1 | Tripterygium wilfordii |
| MOL003239 | Triptofordin F2 | Tripterygium wilfordii |
| MOL003241 | Triptofordin F4 | Tripterygium wilfordii |
| MOL003242 | Triptofordinine A2 | Tripterygium wilfordii |
| MOL003244 | Triptonide | Tripterygium wilfordii |
| MOL003245 | Triptonoditerpenic acid | Tripterygium wilfordii |
| MOL003248 | Triptonoterpene | Tripterygium wilfordii |
| MOL003266 | 21-Hydroxy-30-norhopan-22-one | Tripterygium wilfordii |
| MOL003267 | Wilformine | Tripterygium wilfordii |
| MOL003278 | salaspermic acid | Tripterygium wilfordii |
| MOL003279 | 99694-86-7 | Tripterygium wilfordii |
| MOL003280 | TRIPTONOLIDE | Tripterygium wilfordii |
| MOL000358 | beta-sitosterol | Tripterygium wilfordii |
| MOL000211 | Mairin | Tripterygium wilfordii |
| MOL000422 | kaempferol | Tripterygium wilfordii |
| MOL000449 | Stigmasterol | Tripterygium wilfordii |
| MOL002058 | 40957-99-1 | Tripterygium wilfordii |
| MOL003283 | (2R,3R,4S)-4-(4-hydroxy-3-methoxy-phenyl)-7-methoxy-2,3-dimethylol-tetralin-6-ol | Tripterygium wilfordii |
| MOL004443 | Zhebeiresinol | Tripterygium wilfordii |
| MOL005828 | nobiletin | Tripterygium wilfordii |
| MOL007415 | [(2S)-2-[[(2S)-2-(benzoylamino)-3-phenylpropanoyl]amino]-3-phenylpropyl] acetate | Tripterygium wilfordii |
| MOL007535 | (5S,8S,9S,10R,13R,14S,17R)-17-[(1R,4R)-4-ethyl-1,5-dimethylhexyl]-10,13-dimethyl-2,4,5,7,8,9,11,12,14,15,16,17-dodecahydro-1H-cyclopenta[a]phenanthrene-3,6-dione | Tripterygium wilfordii |
| MOL009386 | 3,3'-bis-(3,4-dihydro-4-hydroxy-6-methoxy)-2H-1-benzopyran | Tripterygium wilfordii |
| MOL011169 | Peroxyergosterol | Tripterygium wilfordii |
| MOL001323 | Sitosterol alpha1 | Coix Seed |
| MOL001494 | Mandenol | Coix Seed |
| MOL002372 | (6Z,10E,14E,18E)-2,6,10,15,19,23-hexamethyltetracosa-2,6,10,14,18,22-hexaene | Coix Seed |
| MOL002882 | [(2R)-2,3-dihydroxypropyl] (Z)-octadec-9-enoate | Coix Seed |
| MOL000359 | sitosterol | Coix Seed |
| MOL000449 | Stigmasterol | Coix Seed |
| MOL008118 | Coixenolide | Coix Seed |
| MOL008121 | 2-Monoolein | Coix Seed |
| MOL000953 | CLR | Coix Seed |
| MOL002092 | Antioxidant | Centipede |
| MOL000042 | LPG | Centipede |
| MOL000953 | CLR | Centipede |
| MOL000860 | stearic | Centipede |
| MOL000061 | Prolinum | Centipede |
| MOL007355 | acetylcholine | Centipede |
| MOL001464 | histamine | Centipede |
| MOL001831 | HX | Centipede |
| MOL001841 | indole-3-acetic acid | Centipede |
| MOL001744 | uracil | Centipede |
| MOL000114 | vanillic | Centipede |
| MOL000067 | L-Valin | Centipede |
