# Supplementary material for: Exploration of the Immuno-Inflammatory Potential Targets of Xinfeng Capsule in Patients with Ankylosing Spondylitis Based on Data Mining, Network Pharmacology, and Molecular Docking
Source: Evid Based Complement Alternat Med. 2022 Mar 23;2022:5382607. doi: 10.1155/2022/5382607 (PMC8967514; doi:10.1155/2022/5382607)
Supplement: Supplementary Materials — Active ingredients of XFC (Supplementary Table S1) and 57 targets of XFC for AS treatment (Supplementary Table S2) are in Supplemental Files. [file 5382607.f1.zip › 5382607.f1/Supplementary Table S2.docx]

Supplementary Table S2 57 targets of XFC for AS treatment

| Number | Targets |
| --- | --- |
| 1 | TNF |
| 2 | CRP |
| 3 | IL6 |
| 4 | MMP3 |
| 5 | IL10 |
| 6 | CD40LG |
| 7 | IL1B |
| 8 | IL23A |
| 9 | IFNG |
| 10 | IL1A |
| 11 | CCL2 |
| 12 | VEGFA |
| 13 | IL4 |
| 14 | IL2 |
| 15 | F3 |
| 16 | STAT3 |
| 17 | NFKBIA |
| 18 | CXCL8 |
| 19 | CXCL10 |
| 20 | CD14 |
| 21 | MMP1 |
| 22 | PTGS2 |
| 23 | TIMP1 |
| 24 | RELA |
| 25 | NOS2 |
| 26 | ICAM1 |
| 27 | CAT |
| 28 | CYP1A1 |
| 29 | CD274 |
| 30 | PTGS1 |
| 31 | SPP1 |
| 32 | IGFBP3 |
| 33 | MPO |
| 34 | THBD |
| 35 | ESR1 |
| 36 | GSTP1 |
| 37 | NR3C1 |
| 38 | MMP2 |
| 39 | AR |
| 40 | GSK3B |
| 41 | SELP |
| 42 | PON1 |
| 43 | CCR7 |
| 44 | ADRB2 |
| 45 | OPRM1 |
| 46 | SLC6A4 |
| 47 | HTR3A |
| 48 | RUNX2 |
| 49 | CD40 |
| 50 | SELE |
| 51 | PLAU |
| 52 | FOS |
| 53 | DNMT3B |
| 54 | PRKCB |
| 55 | IRF1 |
| 56 | NPEPPS |
| 57 | XDH |
